# Practical Considerations Concerning Preeclampsia Subgroups

**DOI:** 10.3390/jcm14072498

**Published:** 2025-04-06

**Authors:** Peter Tamas, Balint Farkas, Jozsef Betlehem

**Affiliations:** 1 Doctoral School of Health Sciences, University of Pécs, H-7624 Pécs, Hungarybetlehem@etk.pte.hu (J.B.); 2 Department of Obstetrics and Gynecology, School of Medicine, University of Pécs, H-7624 Pécs, Hungary

**Keywords:** placental preeclampsia, maternal preeclampsia, clinical considerations

## Abstract

Preeclampsia is one of the most serious clinical syndromes which can occur during pregnancy. According to our current knowledge, preeclampsia cannot be cured. However, a significant step forward is the recognizing preeclampsia is not a homogenous syndrome, i.e., different pathological events can lead to the hypertension + symptoms of organ damage, occurring in the second half of pregnancy. Clinically, two kinds of preeclampsia can be distinguished. The “classic” placental preeclampsia of immunological origin is characterized by contracted blood volume, fetal growth restriction, and marked alterations in laboratory indices. Patients in this subtype are characteristically young and primiparous. Clinical symptoms appear during the late second or early third trimester and show a quick progression. The outcome in cases of placental preeclampsia is frequently serious. For preventing the most critical conditions, the necessary delivery induction usually results in a preterm newborn. The maternal preeclampsia is associated with high blood volume. The characteristic augmented gestational weight gain is mostly a condition with a multifactorial background; however, obesity seems a critical risk factor. The early clinical symptoms are leg, and then generalized edema; hypertension and proteinuria appear after that. Laboratory abnormalities are rare; even platelet count remains within the normal range. The outcome is usually favorable; however, serious organ edema can lead to eclampsia or placental detachment. In the case of both types—from the name to the therapy—new data worthy of consideration have been created, which also justifies a change in attitude.

## 1. Introduction

Preeclampsia (PE), in its form as hypertension combined with organ deficiency appearing during the second half of gestation, affects about 5–8% of pregnancies. This serious syndrome is responsible for more than 70,000 maternal and cc. 50,000 fetal deaths annually, worldwide [1,2]. Despite intensive research, its treatment is practically limited to reducing high blood pressure and inducing labor [2]. In recent decades, there has been some progress in the management by the introduction of screening tests with Acetylsalicylic acid prophylaxis and improvement in fetal condition assessment. However, recognizing that preeclampsia is essentially two distinct clinical conditions may open up broad possibilities in understanding the syndrome and, in many cases, its treatment and even prevention.

Pioneers in breaking down the homogeneous disease concept of PE were Easterling et al. [3] in 1990 and Xiong et al. [4] in 2000, when, contrary to the orthodox concept of high vascular resistance/low blood volume and fetal growth restriction, they reported low resistance with high blood volume and high fetal birth weight in preeclamptic pregnancies. An important step was taken by Belfort et al. [5], who found not only reduced but also increased cerebral blood flow in preeclamptic females in 1999. Heterogenetic origins of PE were suspected by Ness and Roberts [6] in 1996; however, the two groups of preeclamptic patients, differing in clinical, hemodynamical, and laboratory parameters, were first reported in 2003 [7].

An evaluation regarding 44.220 relevant data of the Medical Birth Registry of Norway further supported the heterogenic origin of preeclampsia, revealing that the prevalence of not only low but also high fetal birth weights was over the average when mothers had been admitted with preeclampsia diagnosis [8].

Today, the two-type-PE concept is fully accepted. The question, whether common reason(s) (e.g., syncytiotrophoblast stress) causes the symptoms in distinct ways, or totally independent facts lay behind the different types of PE, remains to be answered. A distinction of the subtypes is essential in many terms, especially for treatment [9,10]. Based on the clinical picture (fetal condition, high body mass index with high weight gain, and the appearance of edema as the first sign), this is possible (see later!). A quantitatively expressive difference can be determined through hemodynamic examinations [7,11,12] and now utilizing numerous laboratory tests [13,14,15].

In the case of both the placental and the much more common maternal type [16], new aspects worth considering have emerged, which may shed new light on the realm of PE.

## 2. Diagnosis of Preeclampsia

The current suggested definition of preeclampsia takes into account signs of other organ damage or fetal involvement, in the presence of de novo hypertension rather than proteinuria [17]. Hypertension detected before week 20 may refer to chronic, non-gestational hypertension, while proteinuria appearing already during the first half of gestation could be a marker of any renal disease. Establishing the correct diagnosis of PE is not always easy. The well-known “white coat hypertension” is common: systolic blood pressure of up to 160–170 mm Hg can be repeatedly measured without a true hypertensive state. For determining diastolic value, a fifth Korotkoff sound should be considered [2].

Frequent contamination of urine samples with protein and thus false positives can also lead to misdiagnosis. Moreover, the sensitivity of the test strip for detecting proteinuria is only 50% at a significance level of 1+. The correct method is to avoid contamination, to measure protein content in a 24 h urine collection. However, in cases of maternal PE, the daily protein excretion may fluctuate around 0.3 g, which may cause a strange dilemma of PE or not PE.

## 3. Placental Preeclampsia

### 3.1. Denomination

This is the “classic” type, which is also referred to in the literature as hypovolemic, low CO (cardiac output), preterm, severe, with fetal growth restriction, type I, and, most frequently, early-onset PE. This last term was suggested due to the fact that the outcome of preeclamptic pregnancies is much worse when clinical symptoms appear prior to the 34 weeks than when they appear later [18]. This very important and groundbreaking definition unfortunately carries the potential for misunderstanding and sometimes misclassification since the difference between subgroups is not due to the gestational week when clinical symptoms appear, and they could be noticed later than their manifestations. Hypovolemic or low CO expressions refer to an important pathologic feature, while the placental denomination seems the best since it refers to the origin of this type of PE.

### 3.2. Sequence of Symptoms

The first sign of this type of PE is an elevation in blood pressure; reaching 140/90 mmHg is defined as (1) hypertension, which is significant according to the diastolic pressure as well and increases on a daily basis. Organ dysfunction may appear after several days, typically first characterized as (2) proteinuria, since kidneys are sensitive to hypoxia, and detecting urinary protein is simple. Next, the indices of liver and further kidney involvement are observed; however, lowering of the platelet count can precede all (laboratory) changes [19]. Capillary permeability accessibly increases during the final stage of placental PE, and generalized, visible (3) edema appears, resulting in an acute life-threatening condition.

### 3.3. Hypovolemic Hypertension

This type of later preeclampsia manifests itself very early in pregnancy, and its immunological origin is now considered accepted [20,21]. Immunological imbalance between the maternal immune system and the semi-allograft embryo is also reflected in elevated levels of anti-angiogenic agents, such as soluble fms-like tyrosine kinase 1 (sFlt-1), and soluble endoglin, which erase the functions of placental growth factor (PlGF) and transform growth factor β during the initial period of this type of PE [22,23].

Agents from the underperfused placenta (e.g., antiangiogenic factors, free oxygen radicals, activated tumor necrosis factor, fetal cell debris, and microparticle contents) are considered to cause the damage of the protective endothelial glycocalix layer, which leads to a generalized injury of endothelial cells. Markers of endothelial damage (e.g., circulating endothelial cells, soluble vascular cell adhesion molecule 1, E-selectin, and endocan) show elevated levels in placental PE [24,25].

Damaged endothelial cells release the lower amount of vasorelaxant agents (e.g., nitrogen monoxide (NO), prostacyclin, and endothelium-derived hyperpolarizing factor) and secrete increased amounts of vasoconstrictors, such as endothelin-1(ET-1) [26,27]. Additionally, activated platelets release another effective vasoconstrictor, the thromboxane A_2_. So, the reason for the increased blood pressure in placental PE is clearly due to vasoconstriction, secondary to endothelial injury, caused by placental agents; however, a hypovolemia-induced vasopressin effect may also be present [28].

### 3.4. Organ Dysfunction

Previously, vasoconstriction was considered the cause of decreased tissue blood supply. The necessary change in perspective was based on the understanding of changes in microcirculation. The diameter of flexible red blood cells is 7–8 μm, while the capillary diameter is narrower. Slowing down transpassing of red blood cells ensures enough time for CO_2_/O_2_ exchange. Even normal red blood cells cannot pass through a tube if its diameter is less than 2.9 μm [29]. Once endothelial atherosis with fibrin deposition and platelet adhesions critically narrow capillary diameters, blood cells can become trapped. In our case, red blood cell deformability is reduced, and their aggregability increases with damage and marked slowing flow [30,31], promoting the development of thrombotic microangiopathy (TMA) [32,33].

Platelets, by producing immune-modulator molecules and vasoactive agents basically involved in blood coagulation and even participating in infection defense, play a pivotal role in the pathogenesis of placental PE [34,35].

**Figure 1:**
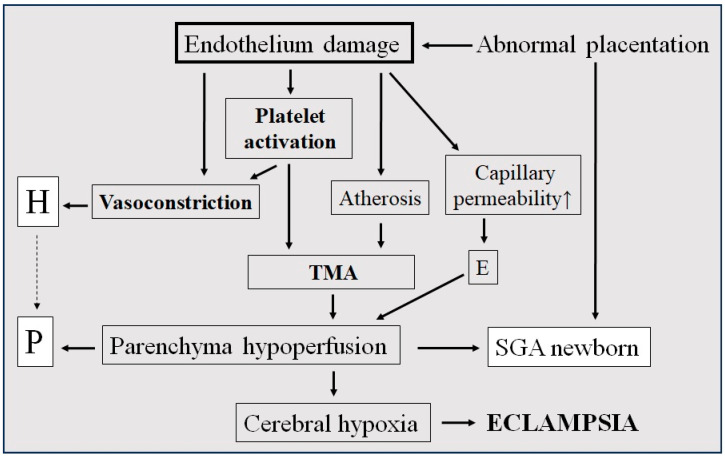
Development of hypovolemic preeclampsia. H: hypertonia, P: proteinuria, E: edema, TMA: thrombotic microangiopathy, and SGA: small for gestational age.

Capillary plugs obviously reduce organ perfusion and increase circulatory resistance. Some entrapped red blood cells break down augmenting lactate dehydrogenase levels; cell fragments can be detected in the bloodstream as signs of peripheral mechanical hemolysis [36]. Adenosine–diphosphate, also released from damaged red blood cells, further enhances platelet activation, promoting the formation of a vicious cycle. Termination of pregnancy should not be delayed if oligo-anuria or signs of central nervous system involvement develops (Figure 1).

### 3.5. Hemodynamics and Fetal Weight

Normally, the average 5 L of blood increases to nearly 7 L by the third trimester, in which the corresponding CO is 6.4–6.8 L/min [37,38]. The augmentation regarding blood volume, which overtakes vasorelaxation by the 12th week, is regulated basically by placental renin through the angiotensin—aldosterone pathway, which is also facilitated by the increased release of vasopressin in early pregnancy; blood pressure does not change significantly. The blood volume in placental PE is low. In the absence of vasodilation, pregnancy hemodilution is also absent. In addition to vasoconstriction, CO lowered even to 3.5 L/min; the deficit can be detected even before the appearance of clinical symptoms [9,10,39].

A special aspect of placental impairment in this type of PE is the weak trophoblast invasion, resulting in an insufficient spiral artery remodeling. These placenta-supplying vessels remain narrow and contractable [40]. The blood supply provided by the small, underperfused placenta proves too insufficient for normal fetal development; fetal somatic restriction is a sine qua non of placental PE. Critical deterioration of the fetal condition in placental PE usually precedes the development of a preterminal maternal condition.

### 3.6. Prognosis and Prophylaxis

At the feto-maternal interface the placenta and the microvasculature are the most impacted tissues in the latent phase of placental PE. Therefore, the markers of placental function [(placental growth factor—PlGF), sFlt-1, pregnancy-associated plasma protein-A, and placental protein 13 and indices of endothelial function asymmetric dimethylarginine (ADMA), ET-1, and vascular cell adhesion molecule 1] to predict PE have been meticulously investigated. In clinical practice, determination of sFlt-1/PlGF ratio is frequently used [41].

Whilst the assessment of the uterine artery (UtA) ultrasonographic Doppler velocimetry analysis has been considered a reliable and non-invasive procedure to evaluate placental blood supply, for the timing of the PE, screening is standardized for measurements between the 11 and 13 + 6 weeks [42]. Pathologic UtA flow (high pulsatility and resistance index, appearance of “notching”) refers to incomplete remodeling of spiral arteries, which contributes the placental insufficiency characteristically specific to this type of PE [43].

Placental PE is most threatening to those who conceive soon after initiating sexual activity, when the attenuation of the maternal immune system against future foreign proteins is not yet sufficient [44]. Based on this, in young nulliparous women, the use of ultrasound and laboratory tests developed for the prediction of PE is entirely justified. In positive cases, high-dose aspirin (150 mg/day) is indicated from early pregnancy onwards, which delays and alleviates the clinical manifestation of placental PE, primarily by inhibiting platelet activation [45].

### 3.7. Management

According to the simplified formula of the fundamental law regarding central hemodynamics, tissue blood supply is determined by the ratio of (blood) pressure to (circulatory) resistance (perfusion = pressure/resistance); i.e., increasing pressure or decreasing resistance improves tissue blood supply. In this sense, the initial increase in blood pressure, in the form of compensation, helps maintain the decreasing perfusion due to the increasing number of capillary blocks [46]. In our case, the question is whether the use of a vasodilator, which simultaneously reduces vasoconstriction and blood pressure, increases tissue blood supply and improves the condition of the mother or the fetus. Since there are no such data, delaying the deterioration of microcirculation (acetylsalicylic acid) has a beneficial effect, which suggests blocking capillaries is more important than vasoconstriction in shaping peripheral resistance. This also implies, in terms of perfusion, vasoconstriction, by elevating blood pressure, has a more beneficial than detrimental effect. Therefore, it is not surprising that the decrease in blood pressure reduces the fetal weight [47]. A rapid and severe drop in blood pressure can lead to a critical condition for the fetus, especially since antihypertensive drugs cross through the placenta [48]. Maternal and/or fetal tachycardia occurring with antihypertensive treatment may be a circulatory compensation to maintain blood supply rather than a side effect of the given drug.

Despite all the above, maintaining blood pressure around 140–160/90 mmHg is justified according to all recommendations, primarily in order to avoid sudden, critically high values. Since hypertension is caused by vasoconstriction, the first recommended drug administered is a vasodilator nifedipine, but labetalol and methyldopa show similar effectiveness in unselected cases [49]. 

Attempts to improve the pathological processes in cases of fully developed disease (such as heparin, acetylsalicylic acid, fish oil, vitamin E, and sedatives) have not yielded any significant results. Magnesium salt and calcium dobesilate (CAD) can be recommended as adjuvants [50]. CAD can have a beneficial effect on microcirculation by promoting both basal and reactive NO synthesis, improving declined erythrocyte deformability, and reducing platelet aggregation and vascular permeability; in gestational hypertension, CAD decreases blood pressure [51,52]. Other promising drugs for the treatment include pravastatin, metformin, and esomeprazole, all of which reduce the antiangiogenic effect of sFlt-1, which plays a key role in disturbing placental development in this type of PE [53]. Prior to week 34, augmenting fetal pulmonary maturation by corticosteroid is indicated [54].

In the case of young nulliparous women, especially with a positive prognosis, regular home blood pressure monitoring is also justified, and hospital admission is recommended once elevated values are detected. Treatment of placental PE is only acceptable in an institution aptly facilitated for managing premature newborns.

## 4. Maternal Preeclampsia

### 4.1. Denomination

High CO, hypervolemic, term, type II, late onset, mild, and maternal PE actually refer to the same pathology. The latter name is perhaps the best because it sharply distinguishes this condition from the other, placental type.

### 4.2. Order of Symptoms

According to many decades of clinical experience, the first sign of “overfilling” is the (1) leg edema, which can develop into generalized form within a few days. Fluid retention can even raise blood pressure, and (2) hypertension develops. Venous congestion with tissue edema, due to further water retention, can lead to symptoms of organ damage, such as (3) proteinuria, which is usually not severe.

### 4.3. Hypervolemic Hypertension

The cause of hypertension which occurs alongside the hypervolemia-induced edema is also the blood volume exceeding a given vascular capacity, which persists despite vasodilation in this form of PE [9,55].

It is known that overloaded circulation itself affects the function of endothelial cells, which, in our case, can also contribute to an increase in blood pressure [56,57]. This is also indicated by the fact that the level of ADMA, which inhibits the formation of the vasodilator NO, is increased both in early-onset and in the late-onset PE when compared to healthy controls [58].

Several data refer to the crucial role of obesity (Body Mass Index—BMI ≥ 30 kg/m^2^) in the development of hypertension, secondary to increased water retention, among nonpregnant women. Similarly, obesity is associated also with gestational hypertensive conditions and shows a positive correlation to edema development [59]. Not only high pre-pregnancy weight but increased weight gain during pregnancy is also associated with the occurrence of this type of PE [60,61].

In obese women, there is elevated plasminogen activator inhibitor 1 and profibrinogen levels, which, due to increasing blood viscosity, contribute to high blood pressure [62]. Insulin resistance, an inherent characteristic of obesity, is known to induce renal sodium and water retention [63]. Moreover, angiotensinogen, crucial for water retention, is also produced by adipocytes [64]

The levels of Na/K-ATPase (Na+ pump) inhibitors, including the digitalis-like marinobufagenin, are increased by salt intake [65]. Therefore, in addition to obesity with sodium retention, the highly potent vasoconstrictor marinobufagenin, also produced by the placenta, is likely to play a role in the pathogenesis of maternal PE, suggesting a specific approach to the treatment of hypertension with hypervolemia [66].

### 4.4. Organ Dysfunction

**Figure 2:**
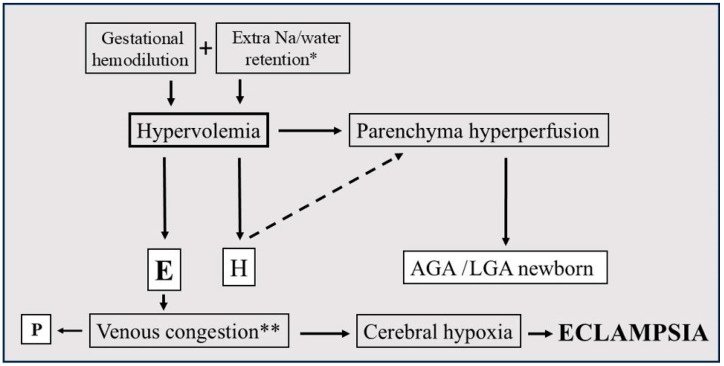
Development of hypervolemic preeclampsia. Asterisk represents * high risk factor: obesity, ** needs further confirmation, H: hypertonia, P: proteinuria, E: edema, AGA: appropriate for gestational age, and LGA: large for gestational age.

Hypervolemia, which ensures favorable tissue blood supply, can cause rapid circulatory deterioration (stasis) when venous outflow obstruction develops [55,67]. Increased renal venous pressure enhances aldosterone secretion, which further increases blood volume and blood pressure [68]. Critical tissue edema can lead to ascites, placental abruption, pulmonary edema, and eclampsia (Figure 2).

### 4.5. Hemodynamics and Fetal Weight

As most names for this PE suggest, in the maternal type, the blood volume is higher than the average for the normal third trimester. In addition to the abundant blood volume (CO—7.5 L/min) and the increased pressure, fetuses usually have an above-average weight [4,51,52]. The relationship between CO and fetal weight has been demonstrated in both healthy and preeclamptic pregnancies [69,70,71,72].

### 4.6. Prognosis and Prophylaxis

Analysis of certain clinical and laboratory data (body weight, weight gain, blood pressure, serum uric acid, creatinine and calcium levels, platelet count, and proteinuria) at the beginning of the third trimester may help predict the expected outcome [73]. Aspirin is not suitable for the prevention of maternal PE [74]. Low-extremity edema or extremely increasing maternal weight gain, as possible first sign of later maternal PE, should be noted during prenatal care. A low-salt and, in some cases, calcium-rich diet may reduce the generalization of edema and the development of hypertension [75,76].

### 4.7. Management

The literature recommends using α- and/or β-blockers as the first drugs recommended in lowering blood pressure [3,77]. It is logical to use a diuretic to treat hypertension which occurs with an overfilled circulation. It is generally accepted, in eclampsia, which means the exacerbation of PE, that diuretic administration is an essential part of management. In spite of that, in PE, diuretics are not generally recommended since they can lead to fatal consequences in the event of hypovolemia. Previous studies have therefore been conducted with diuretics either with prophylactic purposes or administered in the postpartum period of a preeclamptic pregnancy. Collins et al. [78] reported the results of nine studies in 1985. To prevent PE, on the whole, nearly 7.000 pregnant women received continuous administration of thiazid diuretics. This review showed stunningly diverse results and failed to demonstrate reliable evidence of the beneficial effects of diuretics on the prevention of PE. In another examination, twenty-one pregnant women, with elevated CO (considered as pre-hypertensive condition), received a daily administration of 20 mg furosemide, initiated between the thirteenth and thirty-second weeks. An improvement in hyperdynamic circulation was achieved through a significant decrease in stroke volume and CO; however, blood pressure did not drop during the control examinations three weeks later [79]. A randomized placebo-controlled study carried out on preeclamptic patients who received 20 mg of furosemide during the postnatal five days demonstrated that this medication facilitated patient recovery, successfully decreased blood pressure and, in this way, antihypertensive need [80]. In another study, Ascarelly et al. [81] randomly administered 40 mg of furosemide vs. placebo after delivery in 264 patients with severe, mild, or superimposed PE, showing that five days’ medication significantly decreased blood pressure and led to the decreased demand of antihypertensive medication during hospitalization and at discharge in severe preeclamptic patients compared with the other groups. These studies reported overall favorable results. A recent study achieved similar results. In a triple-masked, placebo-controlled, randomized clinical trial, 120 women received 40 mg furosemide or placebo daily following severe PE or eclampsia. In spite of the fact that patients were unselected according to PE types, and the dose of furosemide was fairly low, diuretic medication, on the whole, resulted in a favorable outcome [82].

Importantly, there were no fetal side effects, and neither of the diuretic treatments for gestational hypertension had a negative effect upon perinatal outcome [83].

Considering the increased weight gain during pregnancy, the edema, the hypertension, and even the proteinuria, which all can be explained by water retention beyond the given vascular capacity, the use of diuretics seems to be fully justified in hypervolemic PE. A study on direct diuretic treatment in PE was published in 2017 [84]. In cases with high cardiac output, examined by impedance cardiography, 40 mg of oral furosemide resulted in rapid and parallel reductions in CO and blood pressure. The results confirm the causal role of increased blood volume in the development of hypertension and strongly suggest the need for diuretic treatment in cases of PE associated with high blood volume.

The effectiveness of blood pressure reduction can also be improved by other factors (such as a low-salt and -fat, calcium-rich diet, physical activity, and relaxation techniques) during pregnancy [85]. In addition, mild and careful diuretic treatment (e.g., phytotherapy) can be part of the practice now, without risk, in the case of edema appearing in the third trimester regarding an obese pregnant female.

The termination of pregnancy at the 34–37th gestational weeks of hypervolemic PE is associated with a better maternal, yet a less favorable neonatal, outcome, compared to expectant management [86].

## 5. Conclusions

In summary, we suggest the following considerations for the practice:

- Establishing a correct diagnosis of PE requires proper examination of blood pressure and proteinuria [2].
- Separation of (potential) preeclamptic patients in due time as placental or maternal type is essential since management is also different [9].
- Edema, especially in its generalized form, is a frequent, attention-grabbing sign of imminent maternal PE; obesity is a significant risk factor [59].
- In the setting of developed placental PE, frequent and accurate assessment of fetal status is an essential part of management [87].
- Organ dysfunction is not a consequence of high blood pressure in either type of PE [24,32,67].
- It is worth keeping in mind that a decrease in the blood pressure may influence the fetal condition too, even if an appropriate antihypertensive drug is used [48].
- Following the second trimester, in case of high weight gain and edema regarding an obese female, a mild diuretic treatment may reduce condition worsening [83].
- After delivery, it is important to identify any underlying diseases, which can also help prevent diseases expected in later life, primarily cardiovascular diseases [88].
- The new approach necessitates a modification of the classification of gestational hypertension, which is already being attempted [89,90].

Recognizing that PE is clinically two fundamentally different conditions (they can occasionally mix) explains the previous contradictory research data, greatly helps to understand the pathological events also in relation to individual cases, and may create an opportunity to effectively manage maternal PE. This requires additional laboratory and clinical, bedside studies.

## Data Availability

Data are available upon request from the corresponding author.

## Abbreviations

The following abbreviations are used in this manuscript:

PE	Preeclampsia
CO	Cardiac output
PlGF	Placental growth factor
NO	Nitrogen monoxide
TMA	Thrombotic microangiopathy
ADMA	Asymmetric dimethylarginine
UtA	Uterine artery
CAD	Calcium dobesilate
BMI	Body Mass Index
